# Oil Absorbent Polypropylene Particles Stimulate Biodegradation of Crude Oil by Microbial Consortia

**DOI:** 10.3389/fmicb.2022.853285

**Published:** 2022-05-23

**Authors:** Madalina M. Vita, Paul Iturbe-Espinoza, Matthijs Bonte, Bernd W. Brandt, Martin Braster, David M. Brown, Rob J. M. van Spanning

**Affiliations:** ^1^Systems Biology Lab, Department of Molecular Cell Biology, Faculty of Science, Vrije Universiteit Amsterdam, Amsterdam, Netherlands; ^2^Shell Global Solutions International BV, The Hague, Netherlands; ^3^Department of Preventive Dentistry, Academic Centre for Dentistry Amsterdam (ACTA), University of Amsterdam and Vrije Universiteit Amsterdam, Amsterdam, Netherlands

**Keywords:** biofilm, bioremediation, polypropylene, crude oil, microbial communities

## Abstract

Oil absorbent particles made from surface-modified polypropylene can be used to facilitate the removal of oil from the environment. In this study, we investigated to what extent absorbed oil was biodegraded and how this compared to the biodegradation of oil in water. To do so, we incubated two bacterial communities originating from the Niger Delta, an area subject to frequent oil spills, in the presence and absence of polypropylene particles. One community evolved from untreated soil whereas the second evolved from soil pre-exposed to oil. We observed that the polypropylene particles stimulated the growth of biofilms and enriched species from genera *Mycobacterium*, *Sphingomonas* and *Parvibaculum*. Cultures with polypropylene particles degraded more crude oil than those where the oil was present in suspension regardless of whether they were pre-exposed or not. Moreover, the community pre-exposed to crude oil had a different community structure and degraded more oil than the one from untreated soil. We conclude that the biodegradation rate of crude oil was enhanced by the pre-exposure of the bacterial communities to crude oil and by the use of oil-absorbing polypropylene materials. The data show that bacterial communities in the biofilms growing on the particles have an enhanced degradation capacity for oil.

## Introduction

The Niger Delta extends over about 70,000 km^2^ and has a growing population estimated at 30 million people (Idamoyibo, 2020). In addition, the region has an extensive coastal mangrove forest that plays a critical role in biodiversity and landscape preservation. This habitat has been considerably compromised since the mid-twentieth century when oil extraction from the delta began (Onwuka, 2005; Lindén and Pålsson, 2013). The Nigerian National Oil Spill Detection & Response Agency (NOSDRA) reported that almost 14,000 oil spills occurred in the Niger Delta until 2021 with more than 700,000 barrels of crude oil being dispersed in the environment as a consequence (NOSDRA, 2021). Most of the spills (>90%) were due either to sabotage or theft and illegal oil refining (Brown et al., 2017). Efforts have been made by both SPDC and the Nigerian government to deter illegal activities, and to remediate the area.

Many technologies and approaches are routinely applied in the clean-up of oil spills (Prendergast and Gschwend, 2014). One of these is bioremediation, which relies on microbes to mineralize most hydrocarbons to simpler compounds, and ultimately to biomass, CO_2_ and water. Bioremediation techniques have been extensively studied and applied because they are characterized by cost effective time investments with little disruption of the natural habitat (Bragg et al., 1994; Venosa et al., 1996; Röling et al., 2002; Gogoi et al., 2003; Kostka et al., 2011).

The effectiveness of bioremediation of crude oil depends to a large extent on (i) the supply of electron acceptors (oxidants) to facilitate redox degradation processes, (ii) the bioavailability of the individual oil constituents to the hydrocarbon-degrading microorganisms (Dasgupta et al., 2013) and (iii) whether or not the indigenous microbial community has been pre-exposed to oil, and has adapted to use oil as a food source (Spain et al., 1980).

Another remedial method relies on containment and physical removal of spilled oil using sorbents, e.g., polypropylene. The surface oil is adsorbed or absorbed into the material that is then collected and disposed (Wei et al., 2003; USEPA, 2021). Some remedial technologies combine sorption and biodegradation, for example using sorbents such as chitin, activated carbon or biochar (Setti et al., 1999; Brown et al., 2017). The sorbent material does not only physically remove the oil from the environment, it has the additional advantage of providing a means for optimal attachment of bacteria to form biofilms, enhancing biodegradation (Zellner et al., 1993; Arutchelvi et al., 2011; Lorite et al., 2011). Biofilms are structures with cells embedded in a self-secreted organic polymer matrix that grows on a biotic or abiotic substrate (Costerton et al., 1995; Molin et al., 2000). Bacteria in biofilms have some advantages regarding crude oil degradation compared to their planktonic counterparts. These matrix embedded bacteria show higher biodegradation rates, which are partially attributed to a higher cell loading (Wang et al., 1997; Singh et al., 2006; Han et al., 2020). In addition, biofilms are less likely to be adversely affected by predators, toxins or parasites, in comparison with planktonic cells (Singh et al., 2006; Prabu and Thatheyus, 2007). Moreover, volumetric and topographic analyses performed by Dasgupta et al. (2013) revealed that the *Pseudomonas* biofilms that developed in the presence of crude oil made substantially thicker biofilms compared to those developed in the presence of glucose as the sole carbon and energy source.

We here propose that polypropylene materials can be used not only as sorbents but also as support for enhanced biodegradation. While the effects of sorption on biodegradation has been extensively studied (Mohn, 1997; Chen et al., 2010; Dashti et al., 2017), there is a gap in our understanding of how microbial communities respond to the use of sorbents made of polypropylene, which is a commonly used material in oil spill sorbents. In order to fill this gap, we used passive dosing of crude oil to investigate the effect of an oil-absorbing material on the biodegradation of crude oil by indigenous soil communities of the Niger Delta. Passive dosing is a commonly applied method in toxicity testing of hazardous chemicals and aims at the gradual release of a contaminant from a sorbent material into the surroundings (Oostingh et al., 2015; Bragin et al., 2016). It has been found that such a technique improves biodegradation of alkanes and both homocyclic and heterocyclic PAHs, perhaps through enhanced bioavailability of these molecules for microbes (Humel et al., 2017). More specifically, we rationalized that oil absorption enhances biofilm formation, resulting in increased bioavailability and biodegradation of hydrocarbons. To further elaborate on the community composition in biodegradation, we studied communities with a history of exposure to crude oil at different degrees. Ultimately, we link community pre-adaptation and hydrocarbon availability to the community profiles from biofilms and liquid to display the succession of abundant species during the biodegradation of crude oil. Enhancing biodegradation in sorption media can reduce the amount of hazardous waste which needs to be removed from a site with oil spills. This can have a positive effect on remedial costs on the one hand and reduce negative environmental impacts of hazardous waste disposal on the other hand.

## Methods

### Soils and Crude Oil

The soil and crude oil (Bonny light crude oil) samples used in this study were collected from the Niger Delta as part of a landfarming experiment described by Brown et al. (Brown et al., 2017). One of the soils was left untreated as a control (U) while the other was pre-exposed to oil after which it was biotreated with rhamnolipids (0.5 g/kg) and agricultural fertilizer for 24 days (P) in order to enhance the biodegradation rate of the crude oil (Brown et al., 2017). Soil samples were then shipped to Amsterdam while stored at a low temperature using cooling elements. After arrival, both oil and soil samples were stored at 4°C. Aliquots of the soils were transferred in 2 mL tubes and placed in a freezer at −20°C until the extraction of DNA.

### Growth Conditions

**FIGURE 1:**
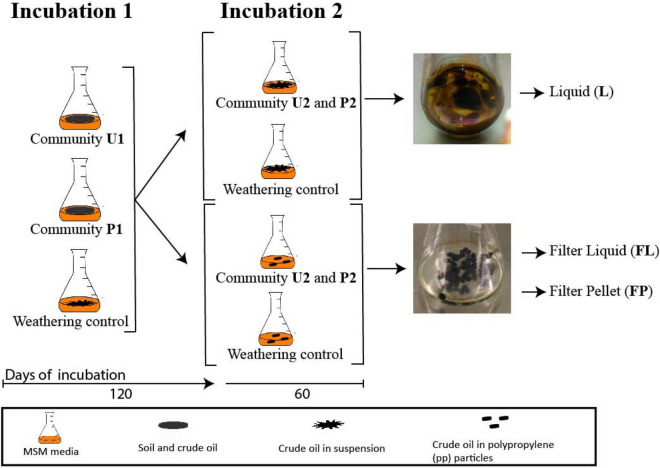
Schematic representation of the experimental design.

The two soil microbial communities (U and P) were cultured in two successive incubations. Incubation 1 lasted 120 days at the end of which aliquots of these cultures were used to start incubation 2, which lasted an additional 60 days, making a total of 180 days. In incubation 1, 2 g of the two soils were added to 250 mL Erlenmeyer flasks containing 47.7 mL of minimum salt medium (MSM; 0.18 mM CaCl_2_, 7.34 mM KH_2_PO_4_, 5.74 mM K_2_HPO_4_, 12.5 mM NH_4_NO_3_ and 1.66 mM MgSO_4_) and 2.5 mL of crude oil as a sole source of carbon and energy and incubated at 120 rpm shaking and 30°C. Incubation 1 will be referred as U1 for the flasks with untreated soil and P1 for the ones with pre-exposed soil, respectively (Figure 1). For Incubation 2, 1 mL aliquots of U1 and P1 cultures were transferred to flasks with fresh media and crude oil and termed U2 and P2, respectively. This second incubation consisted of two parallel experimental conditions. The first one, U2-S and P2-S, reflected the same set-up as incubation 1 where crude oil was added in suspension. The community profiling of these cultures was performed on samples of liquid media. For the second condition, the crude oil was soaked in 60 polypropylene patches of 5 mm in diameter and 2 mm in height (Boso ^®^). The community profiling was performed on the liquid phase of these media (U2-FL and P2-FL) and on detached cells from the polypropylene particles (U2-FP and P2-FP). The amplicon-based composition of both communities U and P was analyzed during U1 and P1, and during U2 and P2 in all phases (Suspended (S), Filter Liquid (FL), and Filter Pellet (FP)). Weathering controls, containing only MSM and crude oil, were included for both conditions to correct for the loss of crude oil at each sampling point. All culturing conditions of incubation 1 and 2 and the weathering controls were set in triplicate. The crude oil contents of both types of culture were determined at the end of incubation 2. A schematic representation of the experimental set-up and sampling scheme can be found in Figure 1.

### Biofilm Detachment From Polypropylene Particles

A protocol for detachment of biofilms from polypropylene particles was described by Mandakhalikar et al. (Mandakhalikar et al., 2018). Briefly, one piece of oil-absorbing material was sampled from the culture flasks at each time point and washed in phosphate-buffered saline (PBS) solution (137 mM NaCl, 2.7 mM KCl, 1.4 mM KH_2_PO_4_, 10 mM Na_2_HPO_4_). After this initial step, the polypropylene particles were transferred to fresh PBS and sonicated at low amplitude for 2 min to suspend and analyze the cells present on the polypropylene matrices. This was repeated four times and for each repetition, the same piece of material was transferred to fresh PBS. The four fractions of PBS obtained in this way were put together in a single tube and centrifuged at 10,000 *g* for 15 min at 4°C. The resulting pellet was resuspended in PBS and stored at −20°C for further analysis.

### Strains Isolation

Culture samples were diluted in PBS, plated on agar plates (1.5%) with Nutrient Broth (NB No. 3, Sigma Aldrich) and incubated at 30°C. After three days, the plates were analyzed for the number and types of colonies. The most abundant colonies, with similar morphology were chosen and isolated by streaking onto fresh NB plates. Isolated strains were stored in glycerol at −80°C for DNA extraction and Sanger sequencing of the 16S rRNA gene.

### DNA Extraction and Identification of Strains

DNA extractions from 250 mg of soil or 200 μL of liquid culture sample were performed using a ZymoBIOMICS DNA Miniprep Kit (Zymo Research, Irvine, CA, United States), according to the manufacturer’s instructions. For the identification of isolates, the almost full-length 16S rRNA gene sequences were amplified using the bacterial specific primers F8 (CAC GGA TCC AGA CTT TGA T(C/T) (A/C) TGG CTC AG) and R1512 (GTG AAG CTT ACG G(C/T) T AGC TTG TTA CGA CTT; Felske et al., 1997). The PCR products were sequenced bidirectionally by the Sanger method (1000 nucleotides nt per read; Macrogen Europe B.V.). Forward and reverse sequences were merged to obtain the complete gene sequence (around 1,500 bp) using the MEGA (v 7) software package. Sequences were compared online to sequences in the nucleotide collection (nt) using megablast (default parameters) on the NCBI BLAST (Basic Local Alignment Search Tool, at https://blast.ncbi.nlm.nih.gov/Blast.cgi; Altschul et al., 1990).

### PCR Amplification and Sequencing

DNA concentrations were measured by the Qubit 3.0 fluorometer (Invitrogen, Life Technologies) using the Qubit 3.0 fluorometer (Invitrogen, Life technologies) and the Qubit dsDNA HS (high sensitivity) kit (Thermo Fischer Scientific, cat. no. Q32851). The PCR amplification (V3–V4 region) and sequencing on the Illumina MiSeq platform (Illumina, San Diego, CA, United States) were done as previously described (Iturbe-Espinoza et al., 2021). The raw sequencing data were deposited in the BioProject database of the NCBI under accession number PRJNA703327.

### Data Analysis

The data analysis of the sequencing results was done as described previously (Iturbe-Espinoza et al., 2021). In short, sequencing reads were processed into an OTU table using USEARCH (Persoon et al., 2017) with some minor modifications: after merging of the paired-end reads, and before clustering, all sequences were additionally quality-filtered using a maximum expected error rate of 0.005 while no ambiguous bases were allowed. Next, sequences passing the maximum expected error rate of 0.002 were clustered into OTUs using the default sequence similarity threshold of 97%. Finally, the sequences passing the 0.005 error threshold were mapped to the cluster centroids to obtain the OTU table. For taxonomic assignments, we used SILVA v 132 (Quast et al., 2012). The SILVA sequences were trimmed to the V3–V4 16S rRNA gene region as described previously (Koopman et al., 2016).

The dataset was subsampled to 10,400 reads per sample. The taxonomic plots are based on relative abundances of the non-subsampled data, after removal of samples not passing the subsampling depth. The subsampled OTU table was used to calculate the alpha diversity indices, OTU Observed (nr of OTUs/sample), and Shannon diversity index using R v 3.6.2 (R Core Team, 2020) and phyloseq v 1.30.0 (McMurdie and Holmes, 2013). Additionally, a principal coordinate analysis (PCoA) plot was created via the function plot_ordination Jensen–Shannon divergence (JSD) to compare the beta-diversity and to uncover clustering of soil samples (using the subsampled OTU table). For the analysis of specific community members, unassigned taxa levels in the OTU table were replaced with their corresponding higher-level taxonomic name. For the generation of the bar plots of the relative abundances, bacterial genera with a relative abundance of less than 0.1% (per sample) were removed. For differential abundance analyses (Log2-fold change) and heatmap plots, only OTUs with a read number higher that 0.1%, over the entire data set were selected. For the differential abundance analyses, the subsampled OTU table was normalized internally with DESeq2 v 1.26.0 using phyloseq (Love et al., 2014). The *p* values were adjusted with the Benjamini and Hochberg correction method (Benjamini and Hochberg, 1995) and an OTU was considered as differentially abundant if its mean proportion was significantly different between sample classes (*p* value < 0.01).

### Crude Oil Extraction and Quantification

Solvent extractable material (SEM) was measured gravimetrically following a liquid-solid extraction with n-hexane according to the USEPA method 1664b (USEPA, 1999). Initially, the cultures were acidified to pH 2 by adding 37% HCl (25 μl/mL) followed by overnight incubation at room temperature. A vacuum system (ABM van Zijl b.v. vacuum pump) was set up for an SPE–Solid-phase extraction, in which the oil molecules present in a sample were adsorbed onto a disk (Empore™ SPE Oil and Grease diam. 47 mm, pk of 20), and subsequently desorbed in n-hexane (Supplementary Figure 1). The n-hexane was evaporated at 60°C until the extract was dry and cooled to room temperature inside a desiccator before weighing. The weight was recorded as the mass per unit volume of oil and grease and reported as n-hexane extractable material (HEM). We statistically compared the oil degraded with and without the polypropylene particles using an unpaired *t*-test in R.

## Results

### Effects of Oil Absorbent Particulates on Biodegradation of Crude Oil

**FIGURE 2:**
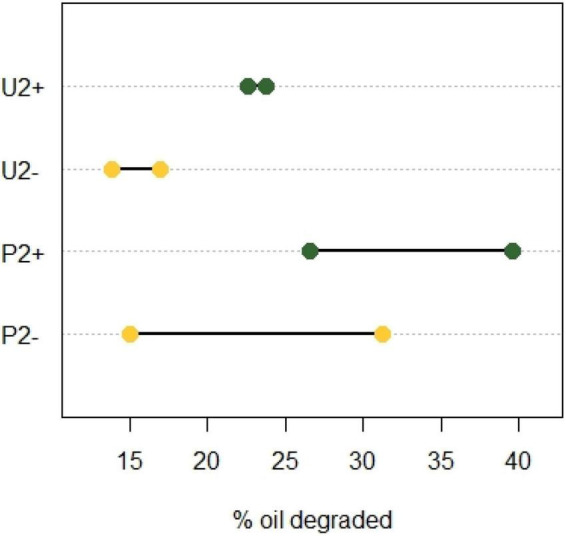
Crude oil degraded in incubation 2 after 2 months with or without polypropylene particles using two enriched soil communities. One from an untreated soil (U) and the other from a soil pre-exposed to crude oil (P). Yellow, cultures without oil absorbent material (−); green, cultures with oil absorbent material (+). The X-axis shows the percentage of oil degraded with respect to the weathering control (*N* = 2).

When observing the mean values of oil degraded at the end of incubation 2, we notice that community P2 had a better oil biodegradation capacity than community U2 (Figure 2). Applying an unpaired t-test revealed that community U+ performed significantly better than community U− (*p* = 0.043, *N* = *2*; Figure 2) showing that the polypropylene particles had a positive effect on the biodegradation of crude oil. Also for communities P, the average performance in biodegradation was higher for the community with polypropylene particles (P +) compared to the one without (P-), but the difference was not statistically significant (*p* = *0.438,N* = *2*).

### Diversity of Oil-Degrading Communities

The DNA extracted from soil and liquid culture samples was used for sequencing the 16S rRNA gene amplicons using the Illumina MiSeq platform. The sequencing resulted in an average of 34,630 reads per sample grouped into operational taxonomic units (OTUs). 94.1% of OTUs were classified. The subsampled OTU table held a total of 320 different genera of which 238 with a relative abundance higher than 0.1% over the entire data set. Most of the OTUs belonged to the phyla *Proteobacteria*, *Firmicutes*, *Actinobacteria*, and *Planctomycetes*.

**FIGURE 3:**
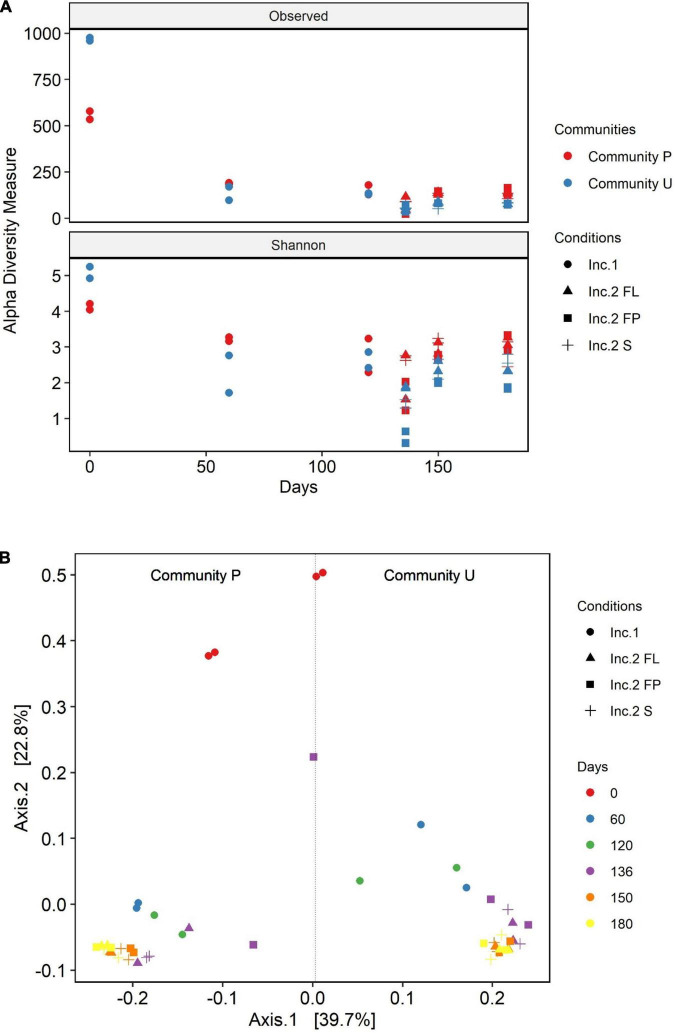
Alpha **(A)** and beta **(B)** diversity analysis of community U and community P. The principal coordinate analysis **(B)** is based on the Jensen–Shannon divergence (JSD) distance. The data are from the sampling points during incubation 1 (Inc.1) day 0 to day 120 and incubation 2 (Inc. 2) for 60 days, which started at experimental day 120 until day 180. The different symbols in both graphs refer to the different incubation conditions: • and ▲ represent the cultures flasks in which the crude oil was added in suspension; ■ and + refer to the cultures in which oil was added soaked in polypropylene particles (pp).

The diversity of the soil communities was analyzed using the alpha and beta diversity indices (Figure 3). Diversity within samples was calculated using the Observed OTU and Shannon diversity indices. Initially, the calculated diversity of community U was higher than the one of community P. At the latest phase of incubation 1, community U underwent a reduction in diversity much more pronounced than that of P. As a result, the two cultures showed similar degrees of diversity at that phase. The presence of the oil absorbent material in incubation 2 caused an additional reduction in the diversity of the microbial communities. The OTUs present in the liquid phase both in the culture with and without the absorbent material have similar alpha diversity values in both communities. The two communities are characterized by a drop-in diversity, 16 days after the switch from incubation 1 to incubation 2. Surprisingly, community P attached to the polypropylene particles had the highest alpha diversity indices in the last sampling point of incubation 2, in contrast with the one of community U, which had the lowest ones. Overall, there was a substantial loss of diversity during the time the communities were halfway in incubation 1 as opposed to the original soil communities. Although there were high differences in richness, the Shannon diversity values were more similar between the two communities.

The PCoA ordination plot of these samples displaying the Jensen–Shannon divergence (JSD) distance revealed that the structure of the communities U and P attached to polypropylene particles are closer to the one in the liquid media than the one in the control flasks without polypropylene particles (Figure 3B). In addition, this index reinforces the fact that the communities are distinct from one another as they group separately. Besides, this plot shows the clear succession of the microbial communities from day zero, incubation 1, and incubation 2. In incubation 2, it was observed differentiation of communities from 136 days with respect to the communities from 150 to 180 days, which are grouped together indicating a stabilization in the dynamic of the microbial communities at the end of the experiment. In addition, in incubation 2, communities from the flasks containing polypropylene particles [attached (FL) or suspended in media (FP)] are more similar to each other than the ones in the flasks without polypropylene particles (S).

### Bacterial Community Shifts

**FIGURE 4:**
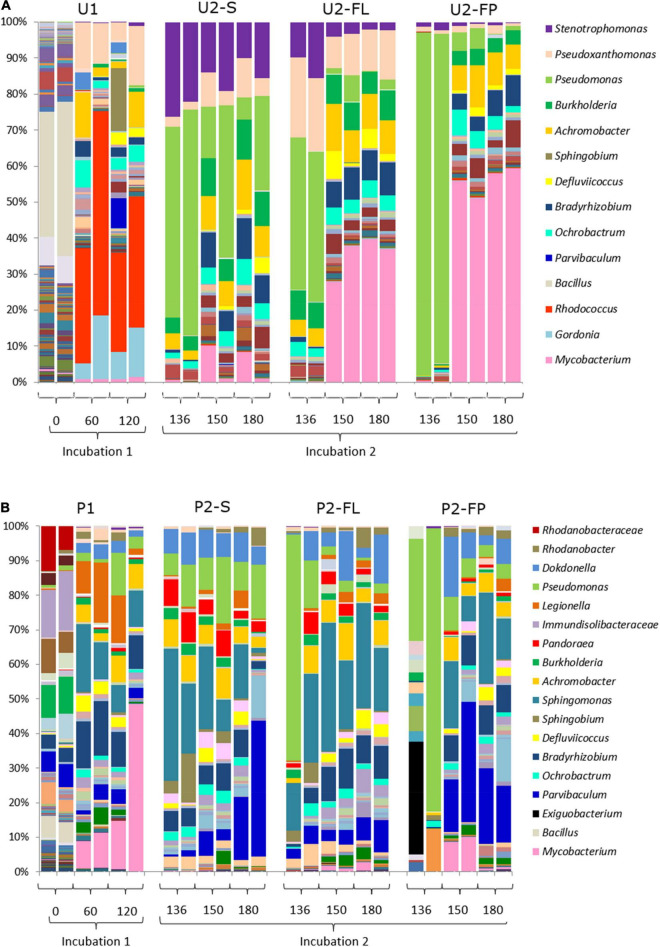
Bacterial community fingerprints. **(A)** Succession of the microbial community from untreated soil (community U); **(B)** Succession of the microbial community from a landfarming experiment (community P). Bar plots are from biological duplicates. Numbers are days. One aliquot of incubation 1 was incubated in two different setups: a medium containing oil in suspension and a medium containing oil absorbed into polypropylene particles (filter-liquid and filter-pellet). Naming legend: 1 and 2 indicate the incubation number, S liquid flasks with oil in suspension, FL liquid from cultures with oil absorbent material (filter liquid), FP biomass detached from polypropylene particles in cultures with oil-absorbing material (filter pellet).

Overall, the amplicon-based composition of community U were distinct from those of community P. Community U1 contained 206 genera with an average abundance higher than 1‰ at the start of the incubation. *Bacillus* was the dominant genus with a relative abundance of 33.6% (Figure 4A). At the same time point, community P had a more even OTU distribution with none of the 136 identified genera contributing more than 13% to the total relative abundance in the original soil (Figure 4B). The incubation with oil in MSM media (U1 and P1) induced the first major shifts in both communities U and P, resulting in an abundance of *Rhodococcus* in community U, and of *Mycobacterium*, *Legionella* and *Sphingomonas* in community P (Figure 4).

The switch to U2 induced a second major change in this community, which remained rather consistent until the end of the incubation period. *Stenotrophomonas* and *Pseudomonas* were the dominant genera after 16 days in U2, although the average contribution of *Pseudomonas* decreased after prolonged incubation (Figure 4A). Community P had a bacterial community composition and dynamics different from those observed for community U. Most notably, *Mycobacterium* was the dominant genus during P1 (Figure 4B), while *Sphingomonas* was the dominant genus in community P2-S. At longer incubation times, *Parvibaculum* became more abundant. In summary, both communities showed major shifts in their composition following changes in the environmental condition, i.e., addition of fresh media and crude oil in incubations 1 and 2. However, although the shifts occurred at the same time they were not the same since the composition of the bacterial communities remained different throughout the incubation, with different genera increasing and decreasing in abundance.

### Selective Effect of Polypropylene Material

The compositions of the communities that developed on the oil absorbent particles (U2-FP and P2-FP) closely reflected those of the liquid media (U2-FL and P2-FL). The presence of polypropylene favored the enhanced proliferation of some genera with respect to the liquid media as well. Overall, both communities U and P were characterized by a strong initial community shift (Figure 4), but they differed in the profiles of the genera and the evenness of their distribution. The abundant presence of the *Pseudomonas* genus after 16 days of cultivation of communities U2-FL, U2-FP, P2-FL and P2-FP was surprisingly characteristic of both communities. The abundance of *Pseudomonas* in community U2-FP became less, while other genera, such as *Mycobacterium*, increased their numbers. The relative abundance of *Mycobacterium* was significantly higher in the cultures with the oil-absorbing material than in the ones without. In P2-FP, a high variability among duplicates was initially present. We attributed this to the rapid shift that occurred when selective pressure was added. From the start of incubation of this community, *Pseudomonas* was outgrown by *Parvibaculum, Sphingomonas* and *Dokdonella*. *Mycobacterium*, dominant in community U2-FP, was present at a relatively high abundance only after 30 days of incubation 2 in community P2-FP. Overall, the community P2-FP had a higher variability and evenness than community U2-FP at the end of the experiment.

### Oil Degrading Genera Selection

**FIGURE 5:**
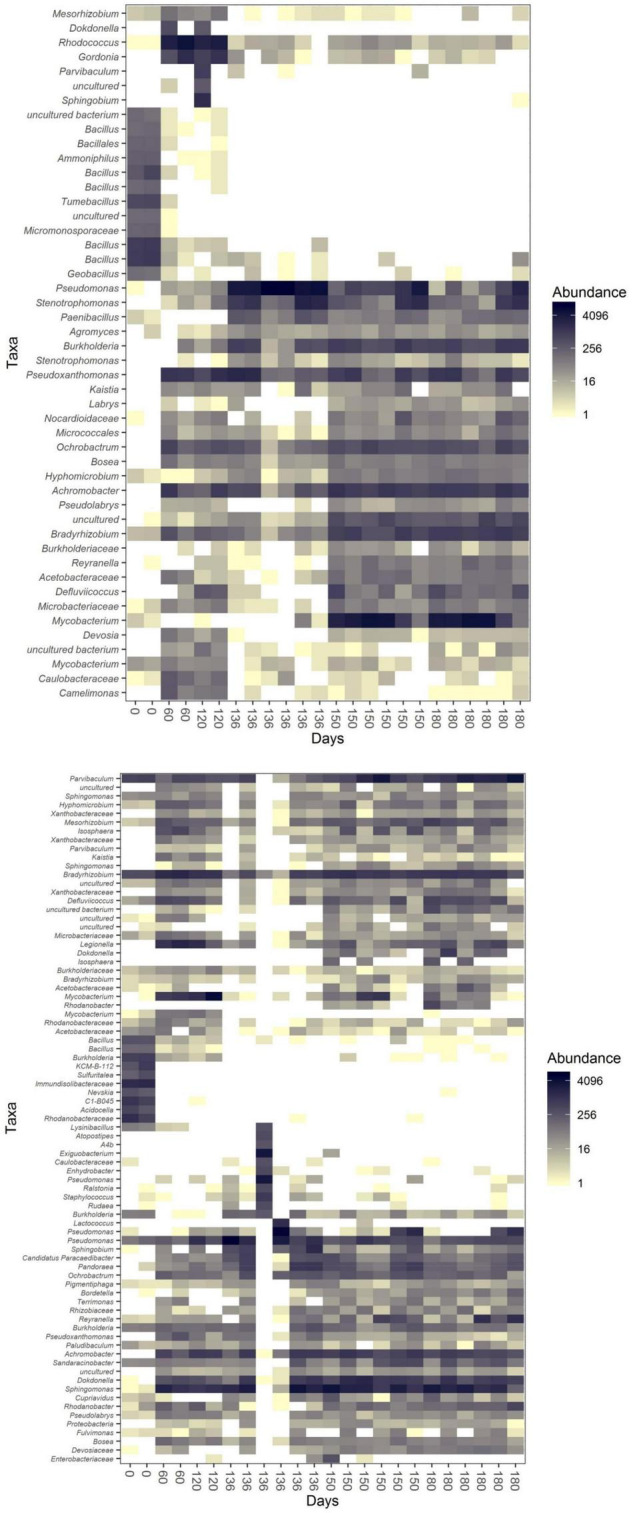
Heatmaps [based on the Jensen–Shannon divergence (JSD) distance] of community U (top) and community P (bottom) during incubation 1 (0–120 days) and incubation 2 (120–180 days of the whole experiment, which correspond to day 0 and 60 days of incubation 2 itself in the data from the subsampled OTU table).

Although the two communities differed in numerous aspects including diversity and composition, they shared several genera. One example is *Achromobacter*, which remained abundant through incubation 1 and 2 but became less abundant during the last weeks of incubation 2 (Figure 5 and Supplementary Figures 2, 3). This pattern was shared by both communities. Similar distribution patterns were seen for *Bradyrhizobium* and *Pseudomonas* as well. The growth of *Pseudomonas*, as discussed above, was highly stimulated by the addition of the oil absorbent material. Its abundance was highest in both communities after 16 days of cultivation of incubation 2.

We observed the succession of the communities upon the incubation switches. *Gordonia* and *Ochrobacrum* were abundant in community U in the first incubation, while *Mycobacterium, Pseudoxanthomonas* and *Strenotrophomonas* became abundant in the second incubation in all three sub-communities (S, FL and FP; Supplementary Figure 2). *Legionella* and *Mycobacterium* were abundant in community P1, while *Dokkdonella, Pandoraea, Parvibaculum, Reyranella* and *Sphingomonas* dominated community P2 (S, FL and FP; Supplementary Figure 3).

**FIGURE 6:**
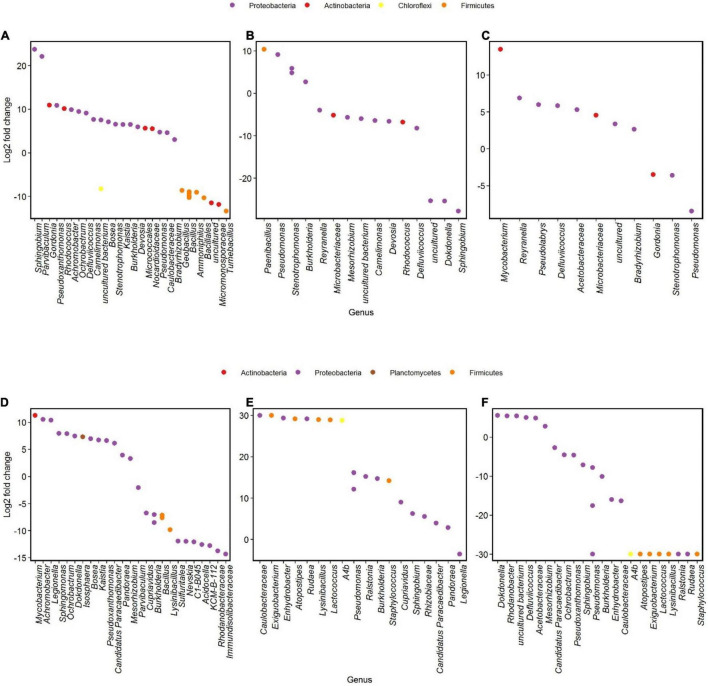
Differential analysis showing OTUs whose abundance significantly changed in time after oil exposure (*p* < 0.01). Log_2_-fold change is the log ratio of observed differences in abundance between two different time points. Genera with Log_2_-fold changes > 0 and < 0 increased and decreased in abundances, respectively. Communities U1 and P1 between days 1 and 120 **(A,D)**, communities U2 and P2 between experimental days 120 and 136 **(B,E**; day 120 of incubation 1 and day 16 of incubation 2, respectively), and communities U2-FP and P2-FP between experimental days 136 and 180 **(C,F**; days 16 and 60 of incubation 2, respectively).

Different genera changed in abundance during incubation 1 and 2 of the two communities. In community U1, genera as *Sphingobium* showed the highest increase between the start and the end of incubation 1, followed by *Parvibaculum* (Figure 6A). We did not notice such increases when we compare the results of the Log_2_-fold changes of genera from community P at the same time points (Figure 6D). Some genera had a significant decrease in Log_2_-fold change during incubation 2, such as *Tumebacillus* and *Micromonosporaceae* in community U2 and *Acidocella* and *Rhodanobacteraceae* in community P2 (Figures 6A,D).

Incubations 2 were always characterized by a major community shift in the first 16 days and minor shifts for the remaining time. During the first part of incubation 2, a member of the *Pseudomonas* genus was abundant in both communities (Figures 6B,E). In between 16 and 60 days of incubation 2, *Pseudomonas* underwent a decrease in both communities, and genera such as *Mycobacterium* and *Dokdonella* were abundant in community U2 (Figure 6C) and in community P2 (Figure 6F), respectively.

### Culturable Strains

During the first incubation culture samples were plated, and the most abundant colonies were isolated for identification by sequencing of the 16S rRNA gene. Most of the isolated strains on the plates were also dominant in the amplicon-based community profiling. Both *Pseudoxanthomonas* and *Rhodococcus* were the dominant genera after 60 and 120 days of incubation 1 in community U. Genera such as *Luteibacter* were identified on plates but not detected by Illumina sequencing. In contrast, *Rhodococcus* was the most abundant genus in the amplicon-based community profiles but not isolated on plates. A full comparison of strains from the abundant colonies on plates and dominating genera in the amplicon-based community profiles at different sampling points can be found in Supplementary Tables 1, 2.

## Discussion

We noticed the formation of biofilms onto the sorbent particles, apparently a suitable substrate for bacterial attachment. The community composition in these biofilms was different from those in cultures with oil in suspension. Bacterial attachment and formation of biofilms on particles during biodegradation was also reported in a study where the presence of activated carbon particles optimized bacteria-driven phenanthrene mineralization (Leglize et al., 2008). Just as seen for our polypropylene disks, the activated carbon particles provided support for biofilm formation and adsorption of the oil molecules. Besides, the role of attached microbes in biodegradation of ethyl *tert*-butyl ether (ETBE) was recently studied in contaminated groundwaters indicating that ETBE-degrading microorganisms preferentially attach to surfaces of sediments (Nicholls et al., 2021). The biofilm-forming bacteria can attach on different types of surfaces through the secretion of exopolymeric substances (EPS). Some evidence has shown that microbial adhesion strongly depends on the extent of hydrophobicity of interacting surfaces (Liu et al., 2004). The same study links a high cell-hydrophobicity to a better microbial adhesion on both hydrophobic and hydrophilic support surfaces. This applies especially to bacteria that form biofilms on highly hydrophobic substrates such as oil (Nikolopoulou, 2013).

In addition to oil coverage, there is a difference in the surface polarity of the PP particulates and sediment grains. While the first solid materials are hydrophobic, the second ones have a more heterogeneous composition and polarity (von Oepen et al., 1991; Kile et al., 1999). Biofilm formation is influenced by the hydrophobicity of the surface as well by the types of bacterial species attached to these surfaces (Marshall, 1985; Dunne and Michael, 2002). Moreover, the hydrophobicity of biofilms has been linked to a high ratio of protein to carbohydrates of the EPS (Santschi et al., 2020). We, therefore, presume that the biofilms formed on the sediment grains would differ in composition and possible biodegradation capacities of crude oil as opposed to the PP particulates. Further studies on natural substrates coated with crude oil are necessary to evaluate to what extent biofilm formation influences crude oil biodegradation under natural conditions.

In order to control for the amount of crude oil added in the cultures flasks we employed polypropylene particles of identical size and number. However, since the biofilm formed on the surface of the particles at the oil/liquid interphase we anticipate that similar results could be achieved with PP sheets that could be fixed on supports and easily recovered at the end of the biodegradation process without being dispersed in the environment. We did not observe any alteration in the breakage of the polypropylene particles after the incubation period and sonication steps. A possible up-scale of this experiment can be performed by using polypropylene sheets in 10–20 L basins where oil is added in suspension or soaked into the polypropylene sheet with a prolonged incubation period of 6 months. In addition, we would advise a synthetic oil with a defined alkane and PAHs composition. By filtering the liquid at the end of incubation, we could determine if the polypropylene sheets are prone to degradation and release of particles in the environment. Moreover, analysis of the crude oil composition will confirm the correlation between the oil composition and the bacterial community composition. We expect the cost-efficiency of this approach to be lower than applying sorbent material alone. The efficiency and cost of this method have been analyzed in multiple studies (Bayat et al., 2005; Sabir, 2015). If most to all toxic crude oil components are biodegraded, the disposal costs of the sorbent will be reduced.

Some bacterial species are more advantaged when it comes to multispecies biofilm formation (An et al., 2006). In our study, some of these species belong to the genus *Pseudomonas*, which was abundant in all communities at the start of incubation 2. Most members of *Pseudomonas* have high motility, which makes it easier for them to actively move to the polypropylene particles and form the first biofilm layers (Thi et al., 2020). Some species such as *Pseudomonas aeruginosa* and *Pseudomonas fluorescens* were reported to form biofilms under almost any growth permissive conditions (O’toole and Kolter, 1998). The analysis of *Pseudomonas* species in the soil communities, which we isolated on agar plates, confirmed the presence and dominance of species related to *P. aeruginosa.* Moreover, members of the genus *Pseudomonas* are known for their ability to degrade both n-alkanes as well as aromatic oil components (Brzeszcz and Kaszycki, 2018).

In the alpha diversity analysis of this study, we observed a reduction of diversity of both communities upon prolonged incubation of the communities with oil. This is in agreement with results from microcosm experiments that showed that oil exposure reduced the diversity of bacterial communities (Röling et al., 2002; Bacosa et al., 2015). This effect has been linked to the disruption of nitrogen cycle processes as a result of the reduction of genes involved in nitrification (van Dorst et al., 2014) and to the cytotoxicity of the oil components or their metabolic intermediates for some species of the bacterial communities (Cerniglia et al., 1983; Hou et al., 2018).

In addition to the presence of oil, the capacity to adhere to the polypropylene particles and form biofilm was an additional selective factor for those genera that grew on the oil absorbent material. As a result, the communities present in the biofilm formed on the polypropylene particles at the end of incubation 2 had the lowest alpha diversity as judged by the Shannon index. Moreover, the abundances of the genera differed between the cultures with or without the particles. This was most profound for *Mycobacterium*, which was abundant in cultures with the particles (Figure 4A). When compared to each other the untreated community U had the lowest number of genera on the particles, although community U was overall less diverse than community P after 60 days of incubation 1. Apparently, the lack of pre-adaptation of this community to crude oil caused a major loss of diversity in the early stages of incubation 1.

The biofilm formed at later stages on the polypropylene particles of community P was enriched in members of the genera *Sphingomonas* and *Parvibaculum*. These genera are often identified as abundant strains in oil-contaminated environments (Paixão et al., 2010; Looper et al., 2013). Bacterial biofilms containing species from these genera have been identified in biofouling studies on reverse osmosis membranes and during bioremediation of persistent organic pollutants (Cerniglia et al., 1983; Looper et al., 2013). This study describes for the first time a stable consortium which degrades natural crude oil. Members of the genus *Mycobacterium* were by far the most abundant microorganisms within the polypropylene-attached community evolved from untreated soil. This is in line with a study where *Mycobacterium* strains selectively grew on PAH-containing hydrophobic membranes (Bastiaens et al., 2000). In addition, various mono- and dioxygenase enzymes important for the degradation of components of crude oil have been identified in various *Mycobacterium* strains (Brezna et al., 2003; Coleman et al., 2011). We could not isolate the mycobacterial species from our community, as they did not show up as abundant colonies on agar plates. They may well be unculturable in isolation or have too low growth rates, which is likely, as many members of this genus are classified as slow growers (Hartmans et al., 2006). Regardless of that, attachment to polypropylene particles most likely induces a niche separation, a situation previously reported in microplastic biodegradation studies as well (Dussud et al., 2018; Miao et al., 2019). In this way, the biofilm offered the environmental stability needed for these mycobacteria to grow at their own pace. This would also explain why the abundance of this genus was lower in the media with suspended oil, most likely due to disruptive effects of continuous shaking of the culture flasks and to losing the competition with fast-growing species.

The composition of the microbial communities in all cultures reflected the changes in crude oil composition regardless of whether the oil was in suspension or soaked in sorbent particles. When adding crude oil, the microorganisms that can use its components and survive in its presence will increase in number. It has been shown that alkanes are degraded faster than the aromatic components of oil (Bacosa et al., 2021). In our work, alkane-degrading bacteria, such as those from the genus *Pseudomonas*, are more abundant during the early stages of culturing. On the other hand, members of the genus *Mycobacterium*, some of which known to contribute to the degradation of complex hydrocarbons, are more abundant at later stages of incubation (Das and Chandran, 2010). Similarly, also *Parvibaculum* was found to be dominant when heavier components of crude oil prevailed the crude oil mixture (Bacosa et al., 2021).

Exposure to crude oil can affect the members of a bacterial community differently. For some strains, the crude oil components can be toxic, thereby inhibiting their growth or killing them. Others can tolerate the presence of oil and may even use the crude oil components as a Gibbs energy and carbon source. These changes contribute to the selection of a community that can degrade or tolerate crude oil. In our study, the microbial community pre-exposed to crude oil degraded higher amounts of the pollutant than the untreated one. This is in agreement with another study where it was observed that soil bacteria pre-exposed to crude oil degraded most of the hydrocarbons at a faster rate than soil bacteria not pre-exposed (Greenwood et al., 2009). The positive effect of pre-exposure on biodegradation has been reported and systematically reviewed for various pollutants, for example, polychlorinated biphenyls and ETBE (Leahy and Colwell, 1990; Poursat et al., 2019, 2020; Nicholls et al., 2020). In our data, the pre-exposed community P had higher variability in community composition and oil degradation. Compared to community U, it degraded more crude oil within 60 days of incubation. Moreover, the decrease in diversity was lower for community P than for community U, indicating that most of the genera were already capable of growth in the presence of crude oil. Possibly, the time to adapt to the oil was shorter for community P than for community U, which may explain the higher degradation rate of community P. Since volatile compounds evaporate rapidly from a crude oil mixture, the community will undergo majors shifts during the initial stages of incubation. This has been observed in both types of incubations where the most dramatic community shifts occurred at the start of the incubations between the first two sampling times. This work reveals that oil absorbed onto polypropylene particles was biodegraded more efficiently than oil in aqueous suspension. In addition, microbial communities are more efficient in the degradation of crude oil when they were pre-exposed to it or when they were collected from oil-contaminated environments. For future environmental oil spills and to prevent the release of small PP particles into the environment, we recommend (i) the use of fixed polypropylene sheets as oil sorbent material and matrix for biofilm formation rather than polypropylene particles, and (ii) the implementation of microbial communities pre-exposed to crude oil. Extrapolation of the data that we have obtained allow us to postulate that the soil microbial diversity increases again after bioremediation is completed and crude oil concentrations are restored to very low levels as a result of that.

## Data Availability Statement

The datasets presented in this study can be found in online repositories. The names of the repository/repositories and accession number(s) can be found below: https://www.ncbi.nlm.nih.gov/, PRJNA703327.

## Author Contributions

All authors listed have made a substantial, direct, and intellectual contribution to the work, and approved it for publication.

## Conflict of Interest

The authors declare that the research was conducted in the absence of any commercial or financial relationships that could be construed as a potential conflict of interest.

## Publisher’s Note

All claims expressed in this article are solely those of the authors and do not necessarily represent those of their affiliated organizations, or those of the publisher, the editors and the reviewers. Any product that may be evaluated in this article, or claim that may be made by its manufacturer, is not guaranteed or endorsed by the publisher.
