# Supplementary material for: Oil Absorbent Polypropylene Particles Stimulate Biodegradation of Crude Oil by Microbial Consortia
Source: Front Microbiol. 2022 May 23;13:853285. doi: 10.3389/fmicb.2022.853285 (PMC9169047; doi:10.3389/fmicb.2022.853285)
Supplement: Supplementary file 1 [file Table_1.DOCX]

# Supplementary Tables

**Table S1**. Isolates of community U after incubation 1. Numbers are relative abundances of the bacterial genera. Grey highlights the genus that has been isolated on agar plates at the sampling time points indicated.

| **Genera** | **Days** | | | | | |
| --- | --- | --- | --- | --- | --- | --- |
|  | 0 | 10 | 30 | 60 | 90 | 120 |
| *Pseudomonas* | 0.000 |  |  | 0.002 |  | 0.004 |
| *Achromobacter* | 0.000 |  |  | 0.074 |  | 0.058 |
| *Bacillus* | 0.379 |  |  | 0.002 |  | 0.001 |
| *Staphylococcus* | 0.000 |  |  | 0.000 |  | 0.001 |
| *Luteibacter* | 0.000 |  |  | 0.000 |  | 0.000 |
| *Stenotrophomonas* | 0.000 |  |  | 0.001 |  | 0.006 |
| *Pseudoxanthomonas* | 0.000 |  |  | 0.118 |  | 0.107 |
| *Rhodococcus* | 0.000 |  |  | 0.443 |  | 0.319 |
| *Ochrobactrum* | 0.000 |  |  | 0.047 |  | 0.039 |
| *Bordetella* | 0.000 |  |  | 0.000 |  | 0.001 |

**Table S2**. Isolates from incubation 1 of community P. Numbers are relative abundances of the bacterial genera. Grey highlights the genus that has been isolated on agar plates at the sampling time points indicated.

| **Genera** | **Days** | | | | | |
| --- | --- | --- | --- | --- | --- | --- |
|  | 0 | 10 | 30 | 60 | 90 | 120 |
| *Pseudomonas* | 0.0110 |  |  | 0.0167 |  | 0.0781 |
| *Achromobacter* | 0.0001 |  |  | 0.0370 |  | 0.0741 |
| *Ochrobactrum* | 0.0000 |  |  | 0.0168 |  | 0.0106 |
