# Supplementary figures and images for: Oil Absorbent Polypropylene Particles Stimulate Biodegradation of Crude Oil by Microbial Consortia
Source: Front Microbiol. 2022 May 23;13:853285. doi: 10.3389/fmicb.2022.853285 (PMC9169047; doi:10.3389/fmicb.2022.853285)

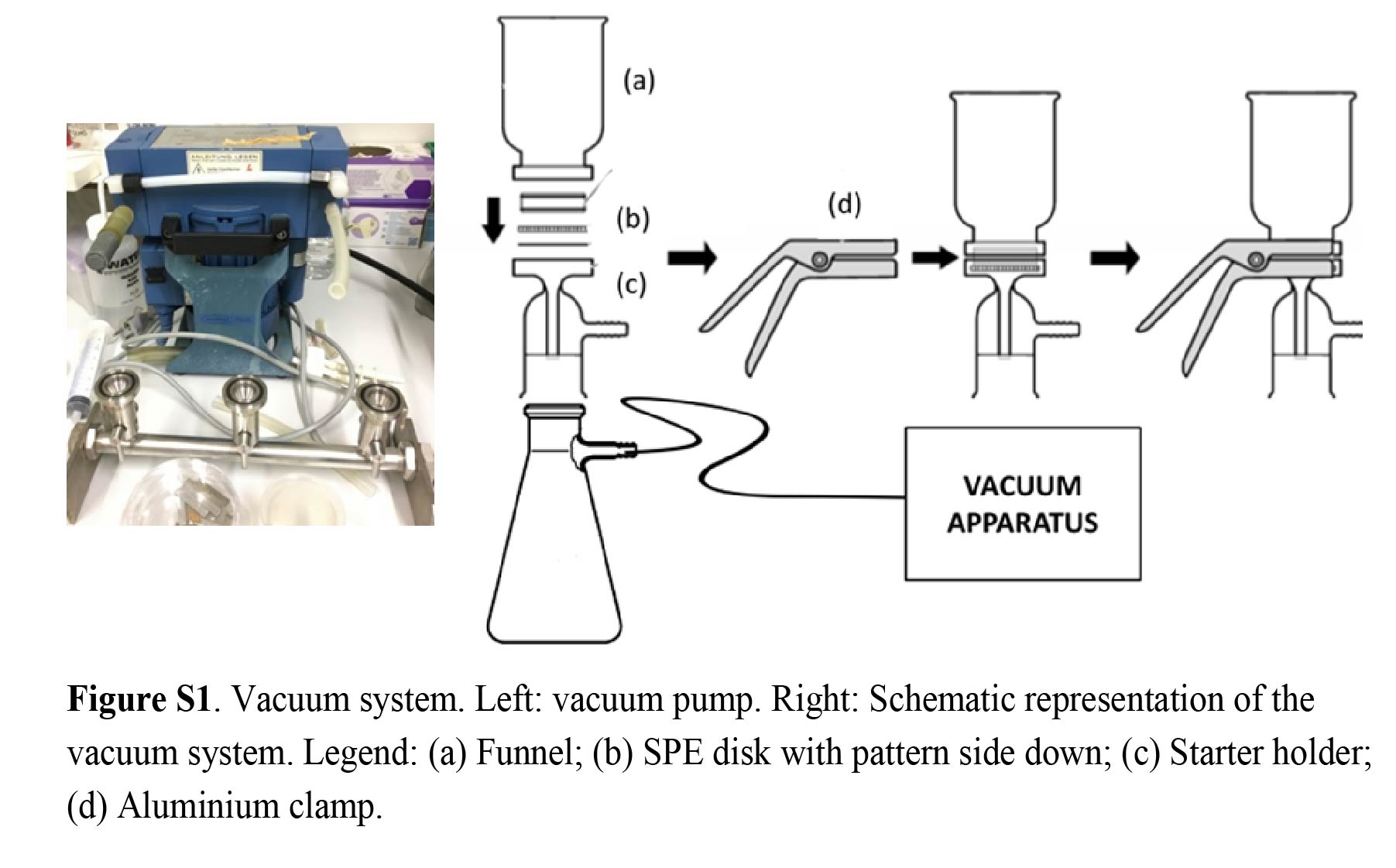

Supplement: Supplementary file 2 [file Image_1.JPEG]

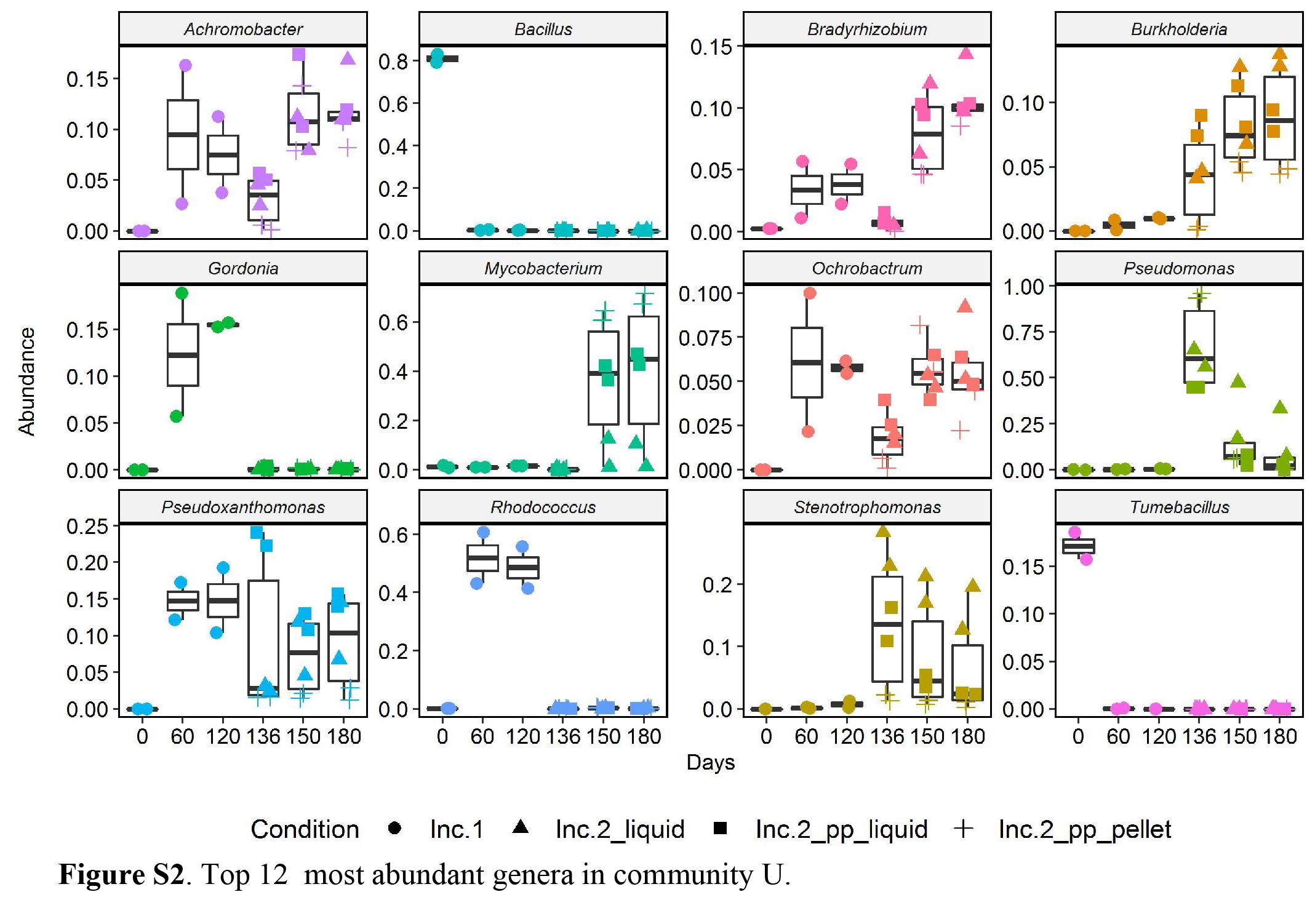

Supplement: Supplementary file 3 [file Image_2.JPEG]

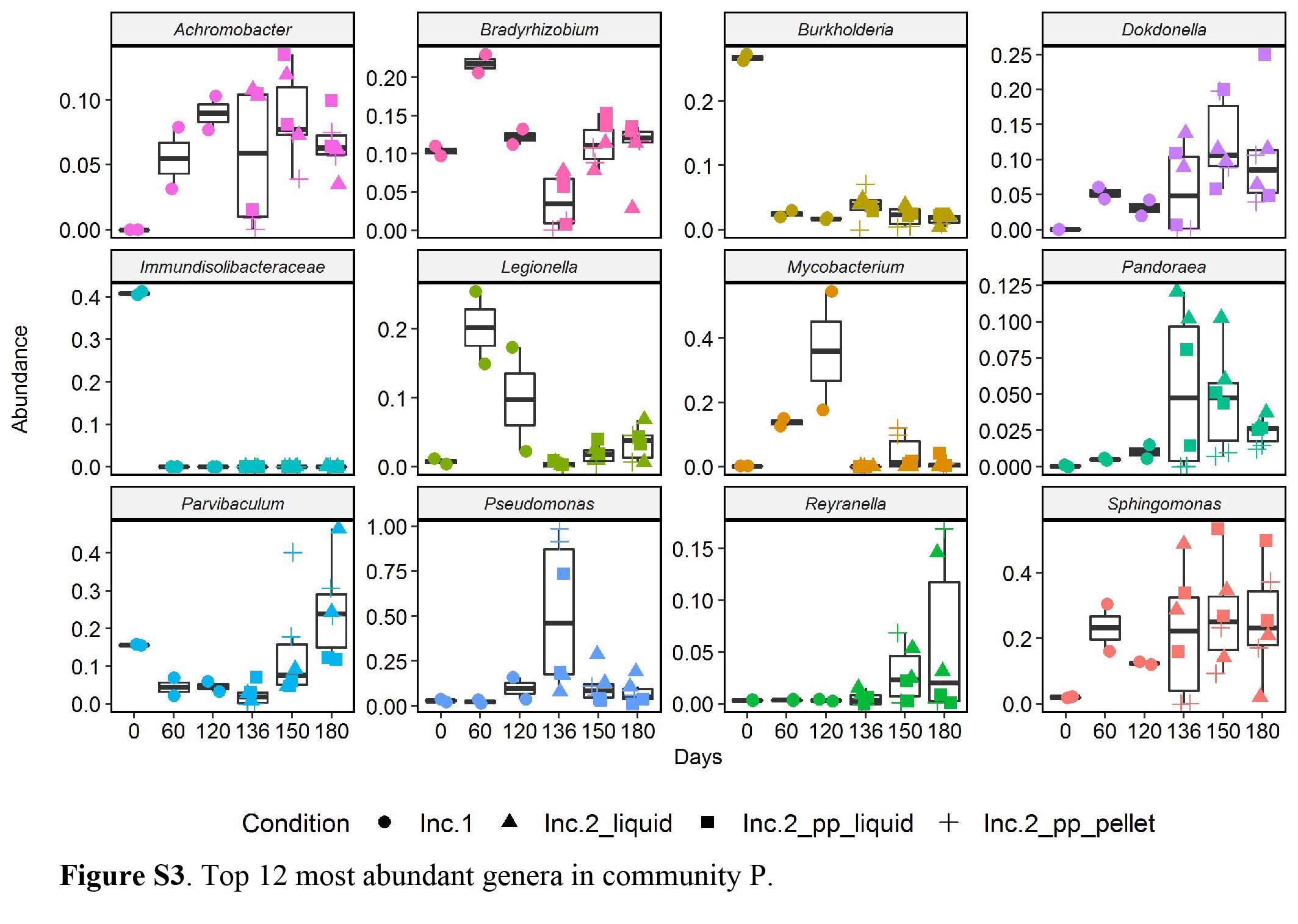

Supplement: Supplementary file 4 [file Image_3.JPEG]
